# Production and In Situ Modification of Bacterial Cellulose Gels in Raisin Side-Stream Extracts Using Nanostructures Carrying Thyme Oil: Their Physicochemical/Textural Characterization and Use as Antimicrobial Cheese Packaging

**DOI:** 10.3390/gels9110859

**Published:** 2023-10-29

**Authors:** Vasiliki Adamopoulou, Anastasia Salvanou, Argyro Bekatorou, Theano Petsi, Agapi Dima, Aris E. Giannakas, Maria Kanellaki

**Affiliations:** 1 Department of Chemistry, University of Patras, 26504 Patras, Greece; adamopoul_v@upatras.gr (V.A.); up1061181@upnet.gr (A.S.); thpetsi@upatras.gr (T.P.); agapidima@upatras.gr (A.D.); m.kanellaki@upatras.gr (M.K.); 2 Department of Food Science and Technology, University of Patras, 30100 Agrinio, Greece; agiannakas@upatras.gr

**Keywords:** bacterial cellulose, raisins, side-stream, nanostructures, thyme oil, *Komagataeibacter sucrofermentans*, textural analysis, antimicrobial food packaging

## Abstract

We report the production of BC gels by *Komagataeibacter sucrofermentans* in synthetic (Hestrin and Schramm; HS) and natural media (raisin finishing side-stream extracts; RFSE), and their in situ modification by natural zeolite (Zt) and activated carbon (AC) nanostructures (NSs) carrying thyme oil (Th). The NS content for optimum BC yield was 0.64 g/L for both Zt-Th (2.56 and 1.47 g BC/L in HS and RFSE, respectively), and AC-Th (1.78 and 0.96 g BC/L in HS and RFSE, respectively). FTIR spectra confirmed the presence of NS and Th in the modified BCs, which, compared to the control, had reduced specific surface area (from 5.7 to 0.2–0.8 m^2^/g), average pore diameter (from 264 to 165–203 Å), cumulative pore volume (from 0.084 to 0.003–0.01 cm^3^/g), crystallinity index (CI) (from 72 to 60–70%), and crystallite size (from 78 to 72–76%). These values (except CI and CS), slightly increased after the use of the BC films as antimicrobial coatings on white cheese for 2 months at 4 °C. Tensile properties analysis showed that the addition of NSs resulted in a decrease of elasticity, tensile strength, and elongation at break values. The best results regarding an antimicrobial effect as cheese coating were obtained in the case of the RFSE/AC-Th BC.

## 1. Introduction

The use of sustainable materials and biomaterials in the food industry is crucial for the development efficient, eco-friendly, and low cost processes. Reflecting this necessity and trend, the scientific community is intensely working on the reduction of food waste and the exploitation of wastes and side streams to produce added value biomaterials, as well as to develop efficient and sustainable processes based on these resources [1,2]. Bacterial cellulose (BC) is one such sustainable biomaterial that can be produced on a variety of natural substrates, such as food wastes, and with applications that include food additives, textiles, cosmetics, medical equipment, and advanced materials. Its structural, physicochemical, and mechanical properties (high biocompatibility, biodegradability, crystallinity, purity, water holding capacity, mechanical strength, moldability, and ability to by synthesized in various forms such as fiber, membrane, hydrogel, etc.), are considered superior to those of plant cellulose, and have been widely studied and reviewed [3,4,5,6,7,8].

BC can be produced by various species, among which *Komagataeibacter* spp. have been established as model microorganisms for its production due to their ability to produce BC of high purity, at high yields, and in a variety of substrates [3,9]. During static fermentation, BC-producing bacteria can form BC hydrogels at the water–air interface, with microstructures ranging from random to ordered [8]. The main drawback of BC production in natural substrates is the low yield, while the production cost in synthetic media is prohibitive. Efforts to produce BC in an efficient and sustainable manner include the use of low cost agri-food wastes as substrates, and various process optimization strategies. Among waste streams that have been successfully used for BC production, the most prominent are crude glycerol, dairy waste (whey), confectionery waste, cereals straw, discarded fruit and fruit processing waste, molasses, etc. [3,6]. Recently, BC was successfully produced by *Κοmagataeibacter sucrofermentans* in aqueous extracts of the industrial raisin finishing side-stream (RFSE), as well as in mixtures of RFSE with cheese whey, aiming to exploit these food industry side streams to add value to their production sectors and support their local economies [3].

BC can form reinforced composites with other materials (biopolymers, nanoparticles, nanoclays, silica, etc.), through in situ and ex situ approaches, that have improved mechanical properties and can be used in a variety of applications such as drug delivery systems, tissue engineering, artificial blood vessels, wound dressings, cosmetics delivery, electronics, antimicrobial films, etc. [8]. In ex situ BC modification, the BC gel is immersed in a solution to interact with the substance of interest (nanostructures, enzymes, proteins, essential oils, etc.), while in the in situ approach, the modification takes places during the fermentation for BC synthesis in the presence of the reinforcing agent, leading to BC composites with improved inherent properties (mechanical properties, crystallinity, porosity, thermal stability, etc.) [6,8,10].

In search of eco-friendly, biodegradable, and sustainable materials, BC and modified BC have been proposed for flexible packaging applications, such as antimicrobial films, an important type of active food packaging (AFP) that is used to preserve the quality of food and extend its shelf life [11]. Many hydrophobic nutrients, such as essential oils (EOs), have been considered as bioactive additives in food and in AFP materials due to their antioxidant and antimicrobial capacities. However, EOs are chemically instable, have low bioavailability, and are insoluble in water, thus limiting their food applications. To overcome these obstacles, various transport systems (carriers) have been developed to protect EOs and control their release [10,12]. Also, since there are several concerns about their safety when directly used in food, their incorporation into AFP films has gained increased attention. To overcome the disadvantage of rapid loss due to their high volatility, the incorporation of EOs into nanostructures as carriers and their subsequent incorporation into AFP polymeric matrices has been proposed [10,12,13,14,15,16,17,18,19]. 

Edible nanoclays, such as natural zeolite (Zt), are nanostructured carriers that have been used to develop bioactive EO–nanoclay nanohybrids. Zts are microporous aluminosilicates, which are excellent adsorbent materials with many applications including biomaterials and foods [18,19], offering possibilities such as for the scavenging of toxic substances, prevention of deteriorative reactions by selective adsorption of oxygen in AFP materials, odor adsorption, antimicrobial properties, and the controlled release of nutraceuticals [18,19]. Activated carbon (AC) has also drawn attention for AFP applications due to its physicochemical properties (uniform porosity, large surface area, low toxicity, surface functionality, etc.), and has been used in combination with nanocellulose to form nanocomposites for AFP applications [20]. 

Natural Zt and AC have been used as nanocarriers for the encapsulation of thyme oil, to develop an AFP for the controlled release of thymol, one of its main constituents [13,14]. The hybrid bioactive zeolite/thyme oil (Zt-Th) nanostructure has been incorporated into a Na-alginate-glycerol polymer matrix, chitosan-polyvinyl alcohol (Ch/PVOH), and low-density polyethylene (LDPE), producing promising films for AFP applications with enhanced tensile, water and oxygen barriers, and antimicrobial properties (against food pathogens such as *Escherichia coli*, *Staphylococcus aureus*, *Listeria monocytogenes*, and *Salmonella enterica*), which could extend the shelf life of meat and fruit products. The incorporation of activated carbon/thyme oil (AC-Th) nanostructures in CS/PVOH and in LDPE produced similar results, increasing the shelf life of meat and fruit products by at least 2 days [13,14].

Based on the above, in order to combine efforts towards sustainable BC production and the development of AFP materials with incorporated nanostructures carrying EOs for the food industry, in this study we report: (a) the production of BC gels by *K. sucrofermentans* and their in situ modification in synthetic Hestrin and Schramm (HS) media and in natural substrates (RFSE), with added Zt-Th and AC-Th nanostructures, (b) the drying and physicochemical/textural characterization of the produced BC films, and (c) their use as antimicrobial AFP materials for white cheese.

## 2. Results and Discussion

### 2.1. BC Yields in Synthetic and Natural Substates with Nanostructures/Thyme Oil

After a set of preliminary experiments for BC production and in situ modification in HS and RFSE substrates with added nanostructures (Zt, Zt-Th, AC, and AC-Th) at various concentrations and at different incubation days (7, 10, and 14 d), the final experimental design that was followed is as shown in Table 1. Since it is well established that thyme oil has antimicrobial activity even at low concentrations (~0.01% *v*/*v*) on both Gram-positive and Gram-negative bacteria [21], its direct addition in the substrates was not studied. Combinations that did not produce BC, or produced BC at a low yield and with undesirable mechanical properties (low tensile strength) were not further evaluated. The first set of experiments was carried out in the synthetic substrate (HS) at a wider range of nanostructure concentrations (0.04–0.64 g/L, on dry weight basis). Based on the best combinations of nanostructure type, nanostructure concentration, and incubation time, the subsequent experiments were carried out in the natural substrate (RFSE) as shown in Table 1. The synthetic HS medium was used for comparison, in order to establish the in situ modification of the BC gels, as well as to investigate the inhibitory effect of thyme oil on the activity of *K. sucrofermentans*.

**Figure 1:**
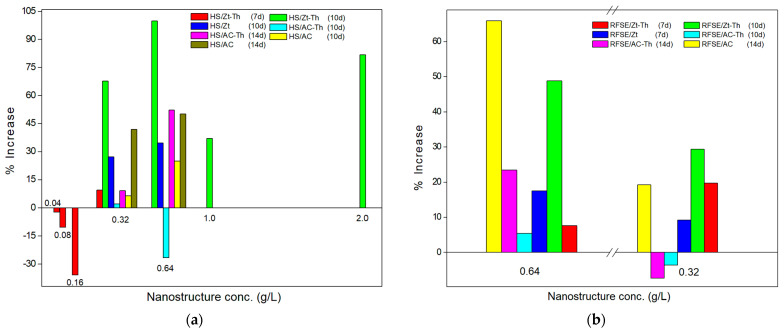
Increase (%) of bacterial cellulose yields at different nanostructure concentrations (dry weight) in the substrates and incubation days. (**a**): Hestrin–Schramm medium (HS) with different concentrations of added zeolite-thyme oil (Zt-Th), or natural zeolite (Zt), or activated carbon-thyme oil (AC-Th), or activated carbon (AC). (**b**) Raisin finishing side-stream extract (RFSE) with different concentrations of Zt-Th, or Zt, or AC-Th, or AC.

Table 2 shows the results of the BC production yields (based on the experimental design of Table 1), while Figure 1 shows the % increase of BC yield compared to the control (no nanostructure added) (relative increase in the mass of the BC/nanostructure composite compared to pure BC).

The lowest concentration of the Zt-Th nanostructure (0.04 g/L) used in the HS medium produced a relatively lower BC yield than the control and the % increase was negative, while when used at 0.32 g/L it led to a ~9% increase in the BC yield. The highest yield in the case of the Zt-Th in the HS medium was obtained at a nanostructure concentration of 0.64 g/L and incubation time of 10 days (2.56 ± 0.12 g/L, 100% increase). In RFSE substrates, the best results were also obtained at a Zt-Th concentration of 0.64 g/L and 7 days incubation (1.67 ± 0.06 g/L, 48.8% increase). Comparing Zt and the Zt-Th addition in both substrates (HS and RFSE), it appears that Zt-Th gave similar or slightly better results. The increased BC yields at the increased nanostructure content, especially with Zt-Th, may possibly indicate a positive effect of the nanostructure on BC production by *K. sucrofermentans,* while the increased thyme oil content did not seem to have an inhibitory effect at these levels.

Regarding the addition of AC-Th, the concentration of 0.64 g/L and 14 days incubation time led to the highest BC yield in both substrates (2.68 ± 0.15 g/L, 52.3% increase in HS, and 2.03 ± 0.04 g/L, 23.4% increase in RFSE). Comparing the results of the AC nanostructures addition in the HS media, it can be observed that AC led to a lower BC yield (2.02 ± 0.04 g/L) than AC-Th, while in RFSE it led to higher yield than that of AC-Th (3.50 ± 0.17 g/L). The increase in incubation time, which was required for the production of BC, is probably due to the addition of AC in the medium, which led to a longer adaptation phase of the bacteria, as other researchers have pointed out [22]. 

In a previous study of BC production in RFSE [3], much higher BC yields were observed, but in that case the RFSE contained higher sugar levels and was fortified by cheese whey or other N-sources (peptone or yeast extract). Specifically, an optimization strategy based on the Response Surface Methodology/Central Composite Design was followed, leading to optimum BC yields of 18.4 g/L in RFSE supplemented with a N-source (at 28 °C, 46.24 g/L initial sugar concentration, and pH 6.42), and 18.9 g/L in RFSE/whey mixtures (at 50.4% whey percentage in the mixture, 1.7% N-source addition, and pH 6.36).

### 2.2. Composition of the Substrates before and after BC Production

To evaluate the level of sugar utilization by *K. sucrofermentans* in the substrates (RFSE) and the organic load of the remaining liquids, the concentration of sugars, organic acids, and Chemical Oxygen Demand (COD), were determined before and after BC production. The results are presented in Table 3. Comparing the composition of the RFSE used in this study (diluted to 4.1 °Be density) with the higher density RFSE reported in [23] (density 11.3 °Be; originating from the same variety of raisins but a different crop year), similar contents (av.) of organic acids were observed (considering the different dilution ratios): citric acid 0.09 and 0.26 g/L, tartaric acid 1.32 and 3.54 g/L, and malic acid 1.43 and 3.94 g/L, respectively. The total (av.) sugars content of RFSE was 8.22% (*w*/*v*), while glucose was found at higher levels (4.36%) than fructose (3.86%) (Table 3). 

In the substrates, after BC production the sugars were reduced, with slightly higher levels (*p* < 0.05) of residual fructose (av. 0.10–0.16%) in all cases except HS, since it did not contain fructose. The residual glucose levels were 0.08–0.14% in the RFSE and 0.06–0.24% in the HS substrates. The utilization of sugars and acids is species-dependent and was possibly affected by the presence of the nanostructures with thyme oil. Specifically, glucose has been reported to be a more preferable energy source for BC-producing bacteria, while organic acids (mainly acetic acid) enhance its production, as shown in several BC optimization studies in synthetic and natural substrates [24]. In this study, the content of organic acids was reduced compared to the original substrate (RFSE). Malic acid presented the highest reduction, followed by citric acid, while tartaric acid showed a smaller decrease. According to [25], citric and malic acid have a synergistic effect on BC production by participating in the carboxylic acid cycle, while tartaric acid possibly has an inhibitory effect on BC production. 

Finally, the COD of the effluents after fermentation varied from 0.7 g/L in the substrates with AC-Th and AC, in both HS and RFSE, to 1.6 g/L in the RFSE with Zt-Th.

### 2.3. Textural Characteristics of the Produced BCs

In Table 4, the textural characteristics of the BC films after drying are presented, as determined via N_2_ adsorption–desorption porosimetry and X-ray diffraction (XRD). The specific surface area (SA) of the control BC produced in RFSE was higher (5.74 m^2^/g) than that of the modified BCs. The modified BC films produced in the HS media had a lower SA (~0.7–0.8 m^2^/g) compared to those produced in HS, as reported in a previous study [3] (Table 4), where the SAs of BC produced in RFSE (20 g/L sugar concentration and 0.5% N-source) and in HS after 7 days of incubation were identical (6.5 m^2^/g) [3]. Furthermore, a reduction was observed in the average pore diameter (APD) (165–190 Å in HS, and 99–138 Å in RFSE) and the cumulative pore volume (CPV) (0.006–0.01 cm^3^/g in HS and 0.003–0.006 cm^3^/g in RFSE) of the BC films containing nanostructures compared to the control samples produced either in HS (201–204 Å and 0.04 cm^3^/g, respectively) [3] or in RFSE (264 Å and 0.084 cm^3^/g, respectively) (Table 4). This reduction is obviously due to the incorporation of the nanostructures into the BC gels, modifying their inherent properties, as was also observed in a study where a single oligosaccharide incorporated into the culture medium reduced the SA, APD, and CPV, as its content in the medium increased [26]. 

Regarding the textural properties of the BC films after 2 months of use as coatings on white cheese (Zt-Th/2 M and AC-Th/2 M), the values appear increased compared to the initial samples (Table 4).

**Figure 2:**
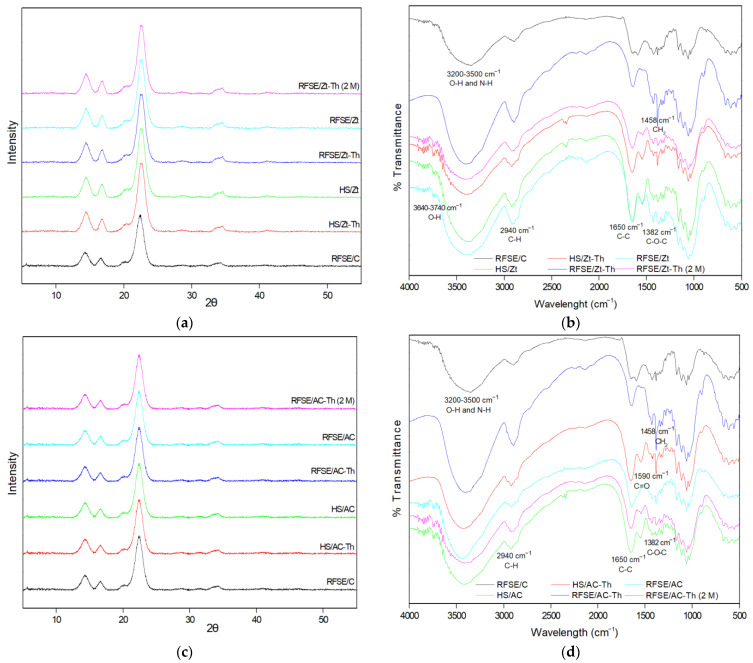
X-ray diffraction spectra (**a**,**c**) and Fourier-transform infrared spectra (**b**,**d**) of bacterial cellulose samples. HS: Hestrin–Schramm medium. RFSE: raisin finishing side-stream extract. Zt: natural zeolite. Zt-Th: zeolite with thyme oil. AC: activated carbon. AC-Th: activated carbon with thyme oil. C: control. M: BC film after 2 months of use as coating on white cheese.

Information about the crystalline structure of the BC films was obtained through the XRD patterns shown in Figure 2a,c. In all cases, three wide peaks at 2*θ* 14.7°, 16.8°, and 22.8° can be seen, characteristic of the cellulose structures Iα and Iβ [3]. Regarding the modified BC films, no peak characteristic of Zt or AC was observed, as was also reported in previous studies. For example, in a film composed of Ch/PVOH and modified with Zt-Th, the characteristic peaks of Zt were not observed, indicating a homogeneous dispersion of modified Zt-Th in the film, which is supported by the Th molecules adsorbed on Zt [27]. Ch/PVOH films modified with AC-Th gave similar results, as the characteristic peaks of AC had almost disappeared [14]. Therefore, there is probably a higher miscibility and dispersion of modified Zt-Th and AC-Th nanostructures in BC [27]. 

As shown in Table 4, the crystallinity index (CI) was lower in the case of the modified BC films (60–70%), compared to the control samples (71–72%). On the other hand, the crystallite size (CS) was similar in both modified and non-modified BC films (72–78 Å), but larger than the CS of the control BC produced in HS (32 Å) [3]. Also, the BC films with Zt-Th and AC-Th after 2 months of use as a coating on white cheese did not present significant differences in their CI and CS compared to the initial BC films.

Figure 2b,d show the FT-IR spectra of dried BC samples with incorporated Zt and AC nanostructures. Absorptions with characteristic peaks at 3300, 2940, 1650, 1458, and 1382 cm^−1^ are attributed to the cellulose groups O-H, N-H, C-H, C-C, CH_2_, and C-O-C, respectively [3,28]. The peak at 1650 cm^−1^ corresponds to the C-C bond and appears more intense in the spectra of the BCs containing Zt-Th and AC-Th due to the aromatic ring of thymol [29]. Also, the peak at 1458 cm^−1^ corresponds to a characteristic peak of thymol and may be related to stretching of the CH_2_ group [30]. This particular peak decreases in the nanostructured BC samples after 2 months of being used as coating on white cheese, which may indicate the release of thymol. 

In all natural Zt-based films, the bands at 3740 and 3640 cm^−1^ are assigned to the OH groups located in the large cavities of the Y-type Zt and suggest the presence of Zt in the obtained films [27]. In the FTIR plot of the BC films containing AC nanostructures there is no characteristic band to suggest the presence of AC in the film. As reported recently, this kind of AC has a broad band at 3430 cm^−1^ corresponding to the OH groups’ vibration and a band at 1590 cm^−1^ corresponding to the carbonyl groups’ vibration [14]. 

In all thyme oil-containing samples, its presence is suggested by the increase of the bands at ~3400 cm^−1^ and at ~3500 cm^−1^, which are assigned to the stretching vibration of the OH groups of thyme oil, and the band at ~1458 cm^−1^, which is assigned to the bending vibration of the CH_2_ group of thyme oil [31]. These two characteristic bands of thyme oil almost disappeared in the case of the films used as cheese coatings and implies the release of thyme oil.

Finally, in the FTIR plots of all modified BC films, no shift peak of the BC bands was obtained, implying the homogeneous dispersion of all nanostructures in the BC matrix [13].

**Figure 3:**
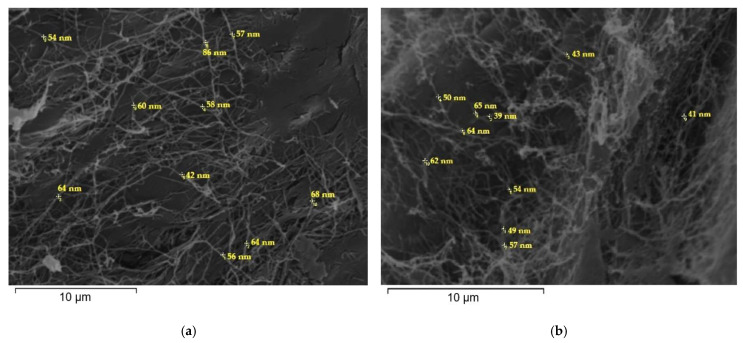
Scanning electron microscopy (SEM) images of dried bacterial cellulose films produced in raisin finishing side-stream extract with incorporated (**a**) natural zeolite-thyme oil (×3000), and (**b**) activated carbon-thyme oil (×3500).

In Figure 3, scanning electron microscopy (SEM) images of dried BC films produced in RFSE with incorporated Zt-Th (a) and AC-Th (b) are presented. BC nanofiber widths of around 42–86 nm (a) and 39–65 nm (b) were measured. Similar BC fiber sizes were previously reported in the case of BCs produced in higher density RFSE (30–70 nm) [3].

### 2.4. Antioxidant Activity of the Modified BC Films

The antioxidant activity (AA) of the dried RFSE/Zt-Th and RFSE/AC-Th BC films, which were used as coatings on white cheese, was determined and compared to the control (RFSE/C). The results were expressed as % absorbance. As shown in Table 4, the control BC film presented the lowest AA (29.4%), while the BC film with AC-Th presented the highest (86.0%). The higher AA is obviously due to the presence of thyme oil in the modified BCs, while differences in AA values indicate different levels of thyme oil retention in the nanostructures and the corresponding BCs. The values determined in the present work, regarding the sample RFSE/Zt-Th (50.8%), are slightly lower compared to the AA reported in the case of the Ch/PVOH/Zt-Th films (49.5–53.5%) [27], while that of the RFSE/AC-Th sample is clearly higher than the value determined in Ch/PVOH/AC-Th films (69.5–72.4%) [14].

### 2.5. Tensile Properties of the BC Films

In Table 4, the values of the modulus of elasticity (Young’s modulus, Ε), the tensile strength (σ_uts_), and the elongation at break (%ε) are presented. Most BC films with integrated nanostructures displayed a decrease in their E value, with the exception of the HS/Zt-Th sample, which showed an increase compared to the control. In terms of σ_uts_, all samples showed a decrease compared to the corresponding control. The %ε increased in most cases, with the exception of samples RFSE/Zt-Th and RFSE/AC, which decreased compared to the control. Lower CI values (~66%) also give lower E (372.5 MPa) and %ε values (15.9) [4]. As concluded from the calculated values of E, σ_uts_, and %ε, the addition of any nanostructures in both substrates resulted in a decrease in both E and σ_uts_. In other words, it could be stated that the addition of nanostructures resulted in more brittle films compared to pure BC films; however, this was not practically perceived when comparing the modified and non-modified BC films. Also, it did not affect the proposed application as AFP, as both modified and non-modified BC films were elastic and easy to apply as coatings on cheese as described below.

### 2.6. Antimicrobial Activity of the BC Films as Coatings on White Cheese

In Table 5, the results of the microbiological analysis of white cheeses coated with BC films with Zt-Th and AC-Th, after 60 days of storage at 4 °C, are presented. White cheese without coating was used as the control. The microbial groups determined were coliforms, enterobacteria, lactobacilli, lactococci, yeasts, molds, and total mesophilic bacteria (TMB). 

In the control sample, an increase in all groups was observed from day 0 to day 60. On the other hand, in the samples coated with the modified BC films, a reduction in the microbial colonies was observed. Specifically, in the white cheese coated with the RFSE/Zt-Th BC, decreased counts of coliforms, enterobacteria, and yeasts and molds were observed until the 21st day, followed by an increase, but at populations remaining at lower levels than those of the control. In the case of lactobacilli, lactococci, and TMB, the reduction was observed until the 14th day. In the cheese sample coated with the RFSE/AC-Th BC, a decrease was observed throughout the storage period, with the exception of coliforms and lactobacilli that remained stable from the 30th to the 60th day, but at low levels.

Similar observations were made by other researchers regarding the antimicrobial effect of Zt-Th composites against foodborne pathogens in vitro [32]. A variety of other thyme oil-carrying AFPs have been used as cheese coatings with similar results, such as sorbitol-amended whey isolate-based film [33], chitosan-based emulsions made of liposomes [17], and chitosan–gelatin-based edible coating fortified with papaya leaves [34], which were effective against foodborne pathogens and maintained microbial counts and physicochemical properties at acceptable levels for at least 4 weeks of chilled storage.

## 3. Conclusions

The results of this study indicate that BC can be modified in situ by addition of Zt and AC nanostructures carrying thyme oil to the growth media of *K. sucrofermentans*, which provide antioxidant and antimicrobial properties to the produced BC gels. The gels can then be dried into films that can be used as AFP. For sustainable BC production, an extract (RFSE) originating from the industrial finishing of premium quality Corinthian currants was used as the substrate. Based on the specific experimental design, the nanostructure content in RFSE for the optimum BC yield was 0.64 g/L for both Zt-Th and AC-Th, yielding 1.67 and 2.03 g BC/L, respectively (Table 2). The textural characteristics of the modified BC films were determined via porosimetry and XRD analysis, showing that the addition of nanostructures, compared to the control, reduced the surface and crystallinity parameters of the BC films in most cases (Table 4). On the other hand, crystallinity was slightly increased after the use of BC films as coatings on cheese. The FT-IR spectra confirmed the presence of the nanostructures and thymol in the modified BCs, while the characteristic bands of thyme oil almost disappeared in the films used as cheese coatings after 2 months (Figure 2). Also, the modified BCs had decreased values of their tensile parameters (Table 4), indicating a more brittle character compared to pure BC films. The Zt-Th and AC-Th BC films gave promising results for use as natural, edible, AFP for cheese. Specifically, the BC/AC-Th film presented better antimicrobial activity as a coating on cheese, and a higher antioxidant capacity (possibly due to the better adsorption of thyme oil on AC and its better integration in the film [15]) (Table 4 and Table 5). The effluent after BC production could be further exploited, as it contains residual sugars, edible nanostructures, thyme oil, and microbial biomass, towards creating added value in the framework of an eco-friendly circular economy. For example, it could be used as a substrate for other biotechnological products within the biorefinery model, as livestock feed, etc., as also reported in a previous study describing a biorefinery for dry wine and baker’s yeast production from RFSE [23]. Future work will focus on such biorefineries, as well as on the optimization of BC production in RFSE, including optimization strategies based on using mixed agri-industrial wastes as substrates. Finally, the development of edible BC-based AFPs with improved mechanical properties will be further investigated.

## 4. Materials and Methods

### 4.1. Chemicals

Alpha-D-Glucose (Serva, Heidelberg, Germany). Yeast extract and 2,2-diphenyl-1-picrylhydrazyl radical (DPPH) (Duchefa Biochemie, Haarlem, The Netherlands). Soy Peptone (Carl Roth, Karlsruhe, Germany). Bacteriological agar, Plate Count Agar (PCA), and M17 agar (Biolab, Zrt., Budapest, Hungary). Calcium carbonate, disodium hydrogen phosphate, and sodium carbonate (Penta, Prague, Czech Republic). Citric acid and acetic acid glacial (Chem-Lab, Zedelgem, Belgium). HPLC-grade tartaric acid, D-fructose, and malic acid (Merck, Darmstadt, Germany). Potato Dextrose Agar (PDA), and MRS agar (Condalab, Madrid, Spain). Ringers solution (tablets; ¼ strength), Violet Red Bile Agar (VRBA), and Violet Red Bile Glucose Agar (VRBGA) (Lab M Ltd., Lancashire, UK).

### 4.2. Materials

The raisin finishing side-stream (RFS) from the industrial standardization of Corinthian currants (premium quality currants; Vostitsa subvariety; Protected Designation of Origin product), was supplied by the Agricultural Cooperatives’ Union of Aeghion S.A. (Aeghion, Greece). RFS is the substandard raisins that cannot be marketed mainly due to the size of the berries (<4 mm, >8 mm). They may also contain damaged berries and stems, as described previously. It is mainly used as raw material for vinegar and alcohol production, and has a high potential for biotechnological utilization due to its high sugar and bioactive nutrients content [3,23]. The aqueous RFS extract (RFSE) was prepared as follows: A quantity of RFS was pasteurized by immersing it in deionized water at 100 °C for 1 min. The RSF was then crushed and pressed manually in fresh water at 70 °C (1:1 RFS to water weight ratio), until an extract of about 80 g/L total sugar content was received (corresponding to a Baumé hydrometer density of ~4 °Be). Hot water was used for the extraction to prevent spoilage or spontaneous fermentation of the extract, and did not exceed 70 °C to avoid sugar degradation reactions. The obtained RFSE was clarified via cloth filtration, pasteurized at 120 °C and 1–1.5 atm, for 1 min, and stored at −18 °C until further use.

The natural ZT, Zt-Th, AC, and AC-Th nanostructures were gifted from the Department of Food Science and Technology of the University of Patras (Agrinio, Greece). In brief, they were prepared as follows: in 2 g of Zt or AC powders, pure thyme oil (Th) was added dropwise under vigorous stirring until homogeneous slurries were obtained. The slurries were stirred for 48 h at 25 °C in open beakers until homogeneous muds were obtained. Then, the Zt/Th and AC/Th muds were sonicated for 12 h and stored until further use. The total amount of thyme oil loaded on natural Zt and AC was calculated gravimetrically and was found to be approx. 37% and 48% (wt), respectively.

The cow’s milk used for white cheese making was a commercial product (KOMIS, Kalavrita, Greece) containing (% *w*/*v*) 3.4 fat, 4.7 carbohydrates, 3.2 protein, and 0.12 calcium.

### 4.3. Microorganism and Nutrient Media

The strain used for BC production was *K. sucrofermentans* DSM 15973 (German Collection of Microorganisms and Cell Cultures, Leibniz Institute DSMZ, Braunschweig, Germany). It was grown at 30 °C for 72 h on a solid nutrient medium (DSMZ, Medium 105) containing (g/L) 10 yeast extract, 100 glucose, 20 CaCO_3_, and 15 agar. The pH was adjusted to 6.0 with the addition of 1 M NaOH solution, and the medium was sterilized at 120 °C for 15 min and at 1–1.5 atm. For the development of the inoculum for BC production, *K. sucrofermentans* from the solid culture was inoculated into 200 mL sterile HS medium containing (g/L) 5 yeast extract, 20 glucose, 5 peptone, 2.7 Na_2_HPO_4_, and 1.15 citric acid. The pH was adjusted to 6.0 with the addition of concentrated acetic acid solution, and inoculation took place at 30 °C for 4 days [3]. The stock culture contained ~0.05 g/mL cell mass (wet weight). All media were sterilized at 120 °C at 1–1.5 atm, for 15 min.

### 4.4. BC Gels’ Production and In Situ Modification in Synthetic and Natural Substates

For the optimization of the production of BC gels in the synthetic (HS) and natural (RFSE) substrates, the following were added to 250 mL conical flasks: (a) 100 mL of sterile HS (pH 6.0) or (b) 100 mL of pasteurized RFSE (pH 3.8). The substrates were left to cool down to 25 °C, and the nanostructure (Zt-Th, Zt, AC-Th, or AC) was added under aseptic conditions and mixed well until an emulsion was obtained. Then, 15 mL of each emulsified substrate were transferred to sterile Petri dishes and 1 mL of the *K. sucrofermentans* stock culture was added under aseptic conditions. The Petri dishes were incubated at 30 °C for 7 to 14 days. The produced BC gels were drained and placed on pre-weighed filter papers, and were oven-dried at 40 °C overnight. Then, they were weighed again to calculate the BC yields, which were expressed as g of BC produced per L of utilized substrate [3]. For the production of dried BC films, for the physicochemical and textural analysis and for use as cheese coatings, the gels were pressed between metal plates covered with non-stick baking paper, and were dried overnight at 40 °C, as shown in Figure S1. The moisture of the BC films after drying at 40 °C (as calculated by further drying at 100 °C until constant weight) was 55.3 ± 2.6% (wt). The moisture of the BC films after use as a coating for 2 months, as described below, was 69.9 ± 1.8%. However, the determined moisture contents at 100 °C may be enlarged due to the evaporation of the thyme oil. It should also be noted that drying at 40 °C produces an elastic film that can be easily applied as a coating on food. Further drying produces a hard, non-elastic film that is not suitable for this purpose and may lead to loss of the essential oil.

### 4.5. Soft White Cheese Making and Coating with BC Films

The soft white cheese was prepared from cow’s milk in the laboratory. In brief, 1 L of milk, heated to 37 °C, was placed in a cubic container and 0.01% *w*/*v* of renin was added and mixed well. The container was placed in an incubator at 37 °C for 1 h. The produced cheese curd was cut into cubes and was placed again at 37 °C for 10 min. Then, the curd was transferred into cubic cheese molds and was left at room temperature (17–23 °C) overnight for whey to drain. A total of 7% *w*/*w* salt was added, and the produced white cheese was kept at room temperature for ~72 h for all the whey to drain.

Amounts of 10 g of the prepared white cheese were placed on Petri dishes and covered with the BC films that were produced in the optimum substrates (RFSE/Zt-Th and RFSE/AC-Th). The films were applied carefully to cover the entire surface of the cheese (Figure S2). The dishes were kept at 4 °C for 2 months and were analyzed for microbial populations as described below. The same sample of white cheese without the BC coating was used as the control.

### 4.6. Analytical Methods

#### 4.6.1. Determination of Sugars and Organic Acids

Sugars and organic acids were analyzed on a LC-2000 Series HPLC system (Jasco Inc., Kyoto, Japan), carrying a Rezex ROA-Organic Acid H+ (8%) LC column (300 × 7.8 mm i.d., 8 μm particle size; Phenomenex, Torrance, CA, USA), a CO-2060 column oven (set at 33 °C), a PU-2089 pump, an AS 2050 PLUS autosampler, and ChromNav 2.0 software. The detectors used were an RI-4030 detector for sugars and a MD-2018 photodiode array detector (at 210 nm) for organic acids. A solution of 0.005 M H_2_SO_4_ (0.5 mL/min) was used as the mobile phase for isocratic separation. Before injection, the samples were filtered through 0.22 µm syringe filters. The injection volume was 100 μL. Analyte concentrations were determined with the aid of standard curves (standard solution concentrations: 0.05, 0.1, 0.5, 1.0, 1.5, and 5% *w*/*v*, for all analytes). Each analysis was performed in triplicate. 

#### 4.6.2. Determination of Chemical Oxygen Demand

The organic load of the liquid residues (effluents) remaining after the production of BC gels was determined via the analysis of COD at a 1:10 dilution. For the analysis, a photometric test was carried out (USEPA Reactor Digestion Method, Hach Lange GmbH, Düsseldorf, Germany). Two mL of the samples (or deionized water for the blank assay) were added at a 45° angle to HACH COD digestion reagent vials (range 0–15,000 ppm). The vials were digested at 150 °C for 2 h (HACH DRB-200 digestion device), cooled to room temperature, and the absorption was measured on a HACH DR/2400 spectrophotometer (at 620 nm).

#### 4.6.3. Textural Characteristics of the Produced BC Films

The analysis of textural characteristics was carried out on the BC gels, produced at the optimum yields in the HS and RFSE substrates with nanostructures, and on the control samples. The samples were dried in an oven dryer at 40 °C overnight and were then ground using a household pepper mill. 

Porosity characteristics (SA, APD, CPV) of the dried BC films (0.1–0.2 g sample; degassed by N_2_ at 95 °C for 120 min) were obtained via N_2_ adsorption/desorption porosimetry, at 77 K and over a wide range of relative pressures using a Micromeritics apparatus (Tristar 3000 porosimeter) [3]. 

For the FT-IR analysis, ~2 mg of sample was mixed with 200 mg KBr (spectroscopic grade) and the mixture was pressed in a hydraulic press (8 ton) for 5 min. The spectra were recorded on a Perkin–Elmer spectrometer (Waltham, MA, USA) (4000–500 cm^−1^, 4 cm^−1^ resolution). Each sample was scanned 10 times [3]. 

The XRD patterns of the BC films were obtained on a Bruker D8 Advance apparatus (Billerica, MA, USA) equipped with a CuKa radiation source that was Ni-filtered. The intensity of diffracted radiation was measured between 5 and 60° (2θ) with a scanning rate of 0.1°/min. The CI was calculated using the Segal equation as described in [3]. 

For SEM, all samples were surface-coated with gold using a Blazers SCD 004 Sputter Coater for 3 min, and studied using a JEOL JSM-5600LV scanning electron microscope (Jeol, Boston, MA, USA).

#### 4.6.4. Tensile Measurements of the BC Films

Tensile measurements were carried out according to the American Society for Testing and Materials (ASTM) D638 method, on a Shimadzu AG-Xplus (5 kNt), instrument (Shimadzu, Kyoto, Japan). Three samples of each film were tensioned at an across-head speed of 10 mm/min. The samples were dumbbell-shaped with gauge dimensions of 31 mm × 13 mm × 0.06 mm. Force (N) and deformation (mm) were recorded during the test. Based on these data and the gauge dimensions, the %ε, σ_uts_, and E values were calculated [13]. 

#### 4.6.5. Antioxidant Activity of the BC Films

The AA assay of the BC films was performed using the DPPH radical scavenging method. Specifically, 50 mg of each film was placed inside a dark bottle and 5 mL of 137.6 μM DPPH methanol solution was added. For all samples, the initial absorbance and the absorbance after 30 min of incubation were measured at 517 nm using a Jasco V-630 UV-vis spectrophotometer (Jasco Inc., Easton, MD, USA). Three samples of each film were measured to obtain the statistical mean as the final measurement [23,27]. The same process was followed without sample addition for the blank assay. The % AC of the active films was calculated by the formula: % AC = [(A_blank_ − A_sample_)/A_blank_] × 100. 

#### 4.6.6. Microbiological Analysis

Representative 10 g of white cheese samples were blended with 90 mL of sterilized Ringer’s solution and subjected to serial decimal dilutions. The following tests were performed for microbiological analysis: (i) coliforms on VRBA at 30 °C for 24 h, (ii) enterobacteria on VRBGA at 37 °C for 24 h, (iii) lactobacilli on acidified MRS agar at 37 °C for 48 h, anaerobically, (iv) mesophilic lactococci on M-17 agar at 30 °C for 48 h, (v) yeasts and molds on PDA at 30 °C for 72 h, and (vi) TMB on PCA at 30 °C for 72 h. Viable counts of the microorganisms were determined in triplicate by plating 0.1 mL (spread plating) or 1 mL (pour plating) of appropriate dilutions on the selective media for each species, under aseptic conditions. The plates containing between 30 and 300 colonies were chosen for enumeration. Determinations took place at 0, 5, 14, 21, 30, and 60 days of storage at 4 °C. The results are presented as log of mean colony forming units per g (log cfu/g).

#### 4.6.7. Statistical Analysis

The significance of the differences in the means of various data groups was checked via a One-Way ANOVA or t-test (one population), at the 0.05 level of significance, using the Microcal™ Origin^®^ software, version 6.0 (Microcal Software, Inc., Northampton, MA, USA). 

## Figures and Tables

**Table 1:** Experimental design for optimization of bacterial cellulose yields in the different substrates with added nanostructures at various concentrations and at different incubation days.

Incubation Time (Days)	NS Concentration in the Substrate (g/L; dry wt)	Type of NS Studied in Each Substrate	
		HS	RFSE
7	0.04	Zt-Th	
	0.08	Zt-Th	
	0.16	Zt-Th	
	0.32	Zt-Th	Zt-Th, Zt
	0.64	Zt-Th	Zt-Th, Zt
10	0.32	Zt-Th, AC-Th, AC	Zt-Th, Zt, AC-Th, AC
	0.64	Zt-Th, AC-Th, AC	Zt-Th, Zt, AC-Th, AC
	1.00	Zt-Th	
	2.00	Zt-Th	
14	0.32	AC-Th, AC	AC-Th, AC
	0.64	AC-Th, AC	AC-Th, AC

NS: nanostructure. HS: Hestrin–Schramm medium. RFSE: raisin finishing side-stream extract. Zt: natural zeolite. Zt-Th: zeolite with thyme oil. AC: activated carbon. AC-Th: activated carbon with thyme oil.

**Table 2:** Experimental BC yields in the different substrates with added nanostructures at various concentrations and at different incubation times.

Substrate	NS Concentration in the Substrate (g/L)	Incubation Time (days)	BC Yield (g/L)				
			C	Zt-Th	Zt	AC-Th	AC
HS	0.04	7	1.72 ± 0.10 ^a^	1.68 ± 0.03 ^a^			
	0.08	7	1.72 ± 0.10 ^a^	1.37 ± 0.13 ^b,c^			
	0.16	7	1.17 ± 0.10 ^c^	0.75 ± 0.11 ^d^			
	0.32	7	1.17 ± 0.10 ^c^	1.28 ± 0.06 ^c,b^			
	0.64	7	1.17 ± 0.10 ^c^	1.32 ± 0.03 ^c,b^			
	0.32	10	1.28 ± 0.06 ^e^	2.14 ± 0.10 ^f,h^			
	0.64	10	1.28 ± 0.06 ^e^	2.56 ± 0.12 ^g^			
	1.0	10	1.28 ± 0.06 ^e^	1.75 ± 0.12 ^f^			
	2.0	10	1.28 ± 0.06 ^e^	2.32 ± 0.10 ^g,h^			
	0.32	10	1.78 ± 0.24 ^f^		2.26 ± 0.12 ^h,i^		
	0.64	10	1.78 ± 0.24 ^f^		2.39 ± 0.04 ^g,i^		
	0.32	10	2.15 ± 0.12 ^j^			2.20 ± 0.02 ^j^	
	0.64	10	2.15 ± 0.12 ^j^			1.58 ± 0.04 ^k,l^	
	0.32	10	1.55 ± 0.02 ^k^				1.65 ± 0.03 ^l^
	0.64	10	1.55 ± 0.02 ^k^				1.94 ± 0.06 ^j^
	0.32	14	1.76 ± 0.25 ^m^			1.92 ± 0.10 ^m^	
	0.64	14	1.76 ± 0.25 ^m^			2.68 ± 0.15 ^n^	
	0.32	14	1.35 ± 0.06 ^m^				1.91 ± 0.02 ^m^
	0.64	14	1.35 ± 0.06 ^m^				2.02 ± 0.04 ^m^
RFSE	0.32	7	1.12 ± 0.08 ^a^	1.45 ± 0.10 ^b^			
	0.64	7	1.12 ± 0.08 ^a^	1.67 ± 0.06 ^c^			
	0.32	7	1.15 ± 0.05 ^a^		1.26 ± 0.05 ^a,d^		
	0.64	7	1.15 ± 0.05 ^a^		1.36 ± 0.05 ^b,d^		
	0.32	10	1.33 ± 0.06 ^e^	1.60 ± 0.05 ^f^			
	0.64	10	1.33 ± 0.06 ^e^	1.43 ± 0.01 ^g^			
	0.32	10	1.55 ± 0.10 ^h^			1.49 ± 0.02 ^h^	
	0.64	10	1.55 ± 0.10 ^h^			1.63 ± 0.03 ^h^	
	0.32	14	1.64 ± 0.13 ^i^			1.52 ± 0.06 ^i^	
	0.64	14	1.64 ± 0.13 ^i^			2.03 ± 0.04 ^j^	
	0.32	14	2.11 ± 0.06 ^j^				2.52 ± 0.13 ^k^
	0.64	14	2.11 ± 0.06 ^j^				3.50 ± 0.17 ^l^

NS: nanostructure. BC: bacterial cellulose. HS: Hestrin–Schramm medium. RFSE: raisin finishing side-stream extract. Zt: natural zeolite. Zt-Th: zeolite with thyme oil. AC: activated carbon. AC-Th: activated carbon with thyme oil. C: control. Superscript letters (a–m for HS, a–l for RFSE) in a row indicate statistical differences between treatments (*p* < 0.05). The statistical analysis was performed for each substrate separately and for each day of incubation (7, 10, 14) between the control and the corresponding nanostructure (C/Zt-Th/Zt and C/AC-Th/AC).

**Table 3:** Composition of the substrates before and after bacterial cellulose production.

Parameter	Initial RFSE	Substrate after BC Production							
		RFSE				HS			
		Zt-Th	Zt	AC-Th	AC	Zt-Th	Zt	AC-Th	AC
Sugars (% *w*/*v*)									
Glucose	4.36 ± 0.07 ^a^	0.11 ± 0.01 ^d^	0.14 ± 0.01 ^e^	0.08 ± 0.01 ^f^	0.14 ± 0.02 ^e^	0.24 ± 0.01 ^b^	0.19 ± 0.02 ^c^	0.06 ± 0.02 ^f^	0.15 ± 0.01 ^e^
Fructose	3.86 ± 0.02 ^a^	0.12 ± 0.02 ^b^	0.16 ± 0.02 ^c^	0.10 ± 0.01 ^b^	0.16 ± 0.01 ^c^	nf	nf	nf	nf
Total	8.22 ± 0.08 ^a^	0.23 ± 0.03 ^b^	0.30 ± 0.03 ^d^	0.18 ± 0.02 ^c,f^	0.30 ± 0.03 ^d^	0.24 ± 0.01 ^b^	0.19 ± 0.02 ^c^	0.06 ± 0.02 ^e^	0.15 ± 0.01 ^f^
Organic acids (g/L)									
Citric	0.09 ± 0.01 ^a^	0.05 ± 0.02 ^c^	0.05 ± 0.01 ^c^	0.05 ± 0.01 ^c^	0.05 ± 0.01 ^c^	1.06 ± 0.03 ^b^	1.09 ± 0.02 ^b^	1.05 ± 0.04 ^b^	1.06 ± 0.04 ^b^
Tartaric	1.32 ± 0.03 ^a^	1.19 ± 0.03 ^b^	1.10 ± 0.03 ^c^	1.17 ± 0.02 ^b^	1.15 ± 0.04 ^b^	nf	nf	nf	nf
Malic	1.43 ± 0.07 ^a^	0.59 ± 0.02 ^b^	0.45 ± 0.04 ^c^	0.58 ± 0.02 ^b^	0.75 ± 0.04 ^d^	nf	nf	nf	nf
COD (g/L)		1.6 ± 0.0 ^c^	1.3 ± 0.0 ^d^	0.7 ± 0.1 ^e^	0.9 ± 0.0 ^f^	1.1 ± 0.1 ^a^	1.2 ± 0.1 ^b^	0.7 ± 0.0 ^e^	0.7 ± 0.1 ^e^

BC: Bacterial cellulose. HS: Hestrin–Schramm medium. RFSE: raisin finishing side-stream extract. Zt: natural zeolite. Zt-Th: zeolite with thyme oil. AC: activated carbon. AC-Th: activated carbon with thyme oil. COD: chemical oxygen demand. nf: not found. Superscript letters in a row indicate statistical differences between treatments (*p* < 0.05).

**Table 4:** Textural characteristics, antioxidant activity, and tensile properties of bacterial cellulose films.

Parameter	Substrate/Nanostructure											
	HS					RFSE						
	C* [3]	Zt-Th	Zt	AC-Th	AC	C	Zt-Th	Zt	Zt-Th/2 M	AC-Th	AC	AC-Th/2 M
Textural properties												
SA (m^2^/g)	6.5–6.0	0.7	0.8	0.7	0.8	5.74	0.5	0.7	0.6	0.2	0.4	0.3
APD (Å)	201–204	190.2	203.1	165.2	174.6	264.4	99.3	110.2	101.1	123.1	138.4	130.9
CPV (cm^3^/g)	0.04	0.006	0.007	0.008	0.01	0.084	0.004	0.006	0.005	0.003	0.005	0.004
CI (%)	70.6–72.4	62.1	59.8	65.6	64.7	72.1	69.0	69.6	69.0	65.0	65.2	65.0
CS (Å)	32.4–31.9	72.7	72.2	75.0	75.7	77.5	72.0	71.7	72	73.0	73.8	73.0
AA (%)						29.4 ± 0.3 ^a^	50.8 ± 0.6 ^b^			86.0 ± 0.3 ^c^		
Tensile properties												
E (MPa)	1737 ± 41 ^a^	1916 ± 26 ^c^	273 ± 54 ^d^	1136 ± 73 ^f^	311 ± 21 ^e^	747 ± 24 ^b^	479 ± 25 ^e^	423 ± 107 ^e^		153 ± 21 ^g^	157 ± 11 ^g^	
σ_uts_	54 ± 11 ^a^	30 ± 13 ^a,b^	29 ± 3 ^c^	26 ± 4 ^c^	21 ± 1 ^c^	34 ± 1 ^b^	17 ± 3 ^c^	25 ± 8 ^b,c^		18 ± 1 ^c^	25 ± 4 ^c^	
%ε	1.8 ± 0.2 ^a^	2.3 ± 0.9 ^a,b^	4.7 ± 1.0 ^b,c^	2.7 ± 0.5 ^a,b^	2.8 ± 0.3 ^a,b^	3.7 ± 0.3 ^b^	3.2 ± 0.2 ^b^	6.2 ± 0.8 ^c^		7.9 ± 1.1 ^c^	1.8 ± 0.3 ^a^	

C*: Data obtained from [3] for comparison (except tensile properties, which are new data). HS: Hestrin–Schramm medium. RFSE: raisin finishing side-stream extract. Zt: natural zeolite. Zt-Th: zeolite with thyme oil. AC: activated carbon. AC-Th: activated carbon with thyme oil. C: control. M: BC film after 2 months of use on white cheese. SA: surface area. APD: average pore diameter. CPV: cumulative pore volume. CI: crystallinity index. CS: crystallite size: AA: antioxidant activity. E: modulus of elasticity (Young’s modulus). σ_uts_: ultimate strength. %ε: elongation at break. Superscript letters in a row indicate statistical differences between treatments (*p* < 0.05).

**Table 5:** Microbial load (log cfu/g) and increase or decrease trend (compared to day 0) of white cheeses coated with BC films with incorporated nanostructures.

Microbial Group	Storage Day	Cheese Sample with BC Coating					
		C	Log cfu/g Trend	RFSE/Zt-Th	Log cfu/g Trend	RFSE/AC-Th	Log cfu/g Trend
Coliforms	0	4.41 ± 0.02	0.00	4.41 ± 0.02	0	4.41 ± 0.02	0
	5	5.33 ± 0.01	0.92	3.77 ± 0.05	−0.64	2.67 ± 0.06	−1.74
	14	5.86 ± 0.03	1.45	3.07 ± 0.06	−1.34	2.46 ± 0.07	−1.95
	21	6.23 ± 0.01	1.82	3.32 ± 0.02	−1.09	2.18 ± 0.03	−2.23
	30	6.47 ± 0.01	2.06	4.31 ± 0.01	−0.1	1.85 ± 0.22	−2.56
	60	6.72 ± 0.01	2.31	4.46 ± 0.01	0.05	1.85 ± 0.13	−2.56
Enterobacteria	0	4.40 ± 0.01	0.00	4.40 ± 0.01	0	4.40 ± 0.01	0
	5	5.37 ± 0.01	0.97	3.33 ± 0.09	−1.07	2.67 ± 0.06	−1.73
	14	6.07 ± 0.02	1.67	2.30 ± 0.07	−2.1	2.07 ± 0.02	−2.33
	21	6.30 ± 0.00	1.90	2.42 ± 0.06	−1.98	2.07 ± 0.02	−2.33
	30	6.47 ± 0.01	2.07	3.45 ± 0.00	−0.95	1.82 ± 0.19	−2.58
	60	6.76 ± 0.01	2.36	4.46 ± 0.01	0.06	1.63 ± 0.06	−2.77
Lactobacilli	0	1.96 ± 0.02	0.00	1.96 ± 0.02	0	1.96 ± 0.02	0
	5	2.26 ± 0.01	0.30	1.93 ± 0.03	−0.03	1.69 ± 0.08	−0.27
	14	2.36 ± 0.00	0.40	0.89 ± 0.19	−1.07	1.14 ± 0.11	−0.82
	21	3.06 ± 0.02	1.10	1.34 ± 0.08	−0.62	0.69 ± 0.09	−1.27
	30	3.18 ± 0.03	1.22	2.04 ± 0.06	0.08	0.63 ± 0.06	−1.33
	60	3.38 ± 0.01	1.42	2.46 ± 0.01	0.5	0.63 ± 0.06	−1.33
Lactococci	0	4.41 ± 0.02	0.00	4.41 ± 0.02	0	4.41 ± 0.02	0
	5	4.60 ± 0.03	0.19	2.51 ± 0.03	−1.9	3.05 ± 0.01	−1.36
	14	5.31 ± 0.02	0.90	2.10 ± 0.02	−2.31	2.06 ± 0.02	−2.35
	21	5.46 ± 0.01	1.05	2.53 ± 0.05	−1.88	1.52 ± 0.01	−2.89
	30	5.92 ± 0.05	1.51	2.95 ± 0.05	−1.46	1.31 ± 0.16	−3.1
	60	6.09 ± 0.01	1.68	3.05 ± 0.02	−1.36	1.04 ± 0.04	−3.37
Yeasts and molds	0	5.29 ± 0.02	0.00	5.29 ± 0.02	0	5.29 ± 0.02	0
	5	5.83 ± 0.05	0.54	3.48 ± 0.05	−1.81	3.62 ± 0.09	−1.67
	14	6.03 ± 0.01	0.74	3.05 ± 0.02	−2.24	3.26 ± 0.05	−2.03
	21	6.28 ± 0.01	0.99	3.03 ± 0.01	−2.26	3.12 ± 0.03	−2.17
	30	6.53 ± 0.02	1.24	3.34 ± 0.14	−1.95	2.62 ± 0.05	−2.67
	60	6.74 ± 0.02	1.45	3.71 ± 0.06	−1.58	2.53 ± 0.05	−2.76
Total mesophilic bacteria	0	5.41 ± 0.01	0.00	5.41 ± 0.01	0	5.41 ± 0.01	0
	5	6.23 ± 0.02	0.82	4.30 ± 0.01	−1.11	4.18 ± 0.03	−1.23
	14	6.31 ± 0.01	0.90	3.88 ± 0.11	−1.53	3.39 ± 0.05	−2.02
	21	6.41 ± 0.02	1.00	4.26 ± 0.03	−1.15	3.28 ± 0.02	−2.13
	30	6.65 ± 0.03	1.24	4.72 ± 0.05	−0.69	2.87 ± 0.03	−2.54
	60	6.95 ± 0.03	1.54	5.07 ± 0.03	−0.34	2.74 ± 0.04	−2.67

BC: bacterial cellulose. HS: Hestrin–Schramm medium. RFSE: raisin finishing side-stream extract. RFSE/Zt-Th: BC produced in RFSE with incorporated natural zeolite with thyme oil. RFSE/AC-Th: BC produced in RFSE with incorporated activated carbon with thyme oil. C: control (no BC coating).

## Data Availability

The data presented in this study are openly available in article.

## Supplementary Materials

The following supporting information can be downloaded at https://www.mdpi.com/article/10.3390/gels9110859/s1, Figure S1. (a) Wet BC gel. (b) Preparation of BC gel for drying (pressed between metal plates covered with non-stick baking paper). (c) Dried BC film; Figure S2. (a) White cheese. (b) Cheese surface covering with dried BC film. (c) Cheese covered with dried BC film after 2 months at 4 °C.
