# Supplementary material for: Production and In Situ Modification of Bacterial Cellulose Gels in Raisin Side-Stream Extracts Using Nanostructures Carrying Thyme Oil: Their Physicochemical/Textural Characterization and Use as Antimicrobial Cheese Packaging
Source: Gels. 2023 Oct 29;9(11):859. doi: 10.3390/gels9110859 (PMC10671232; doi:10.3390/gels9110859)
Supplement: Supplementary file 1 [file gels-09-00859-s001.zip › gels-2639336-supplementary.pdf]

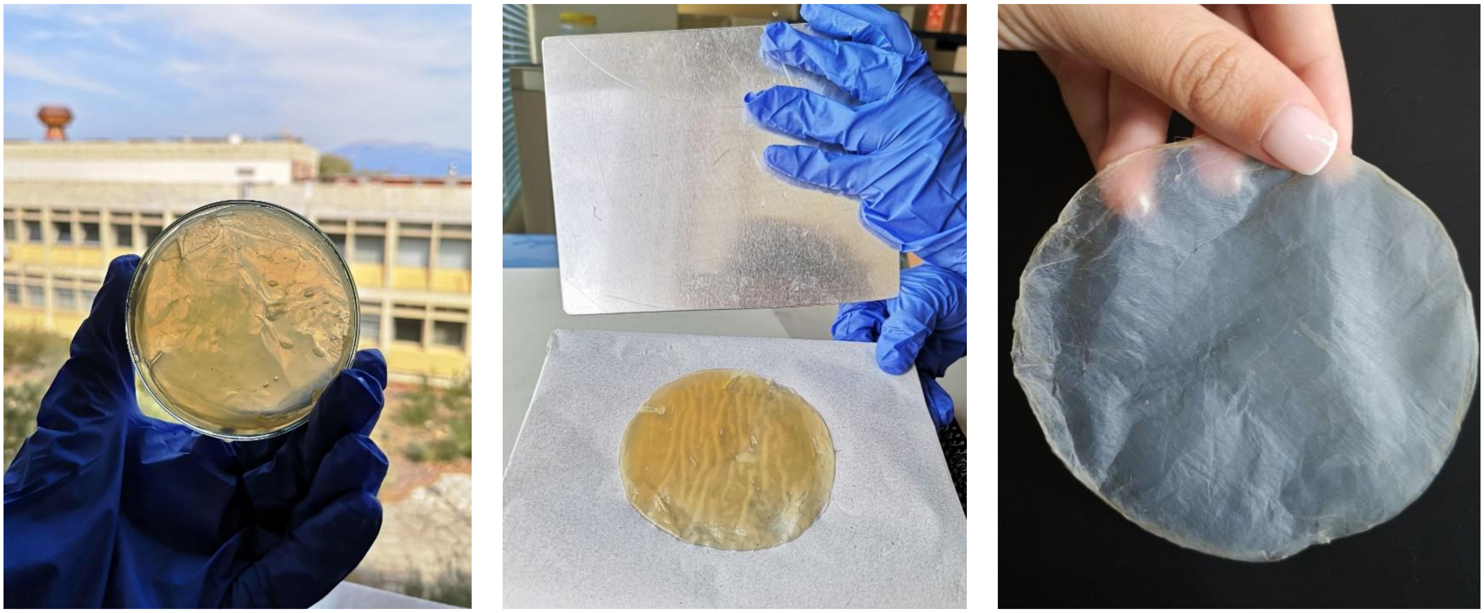

(a) **(b)** **(c)**

Figure S1. (a) Wet BC gel. (b) Preparation of BC gel for drying (pressed between metal plates covered with non-stick baking paper). (c) Dried BC film.

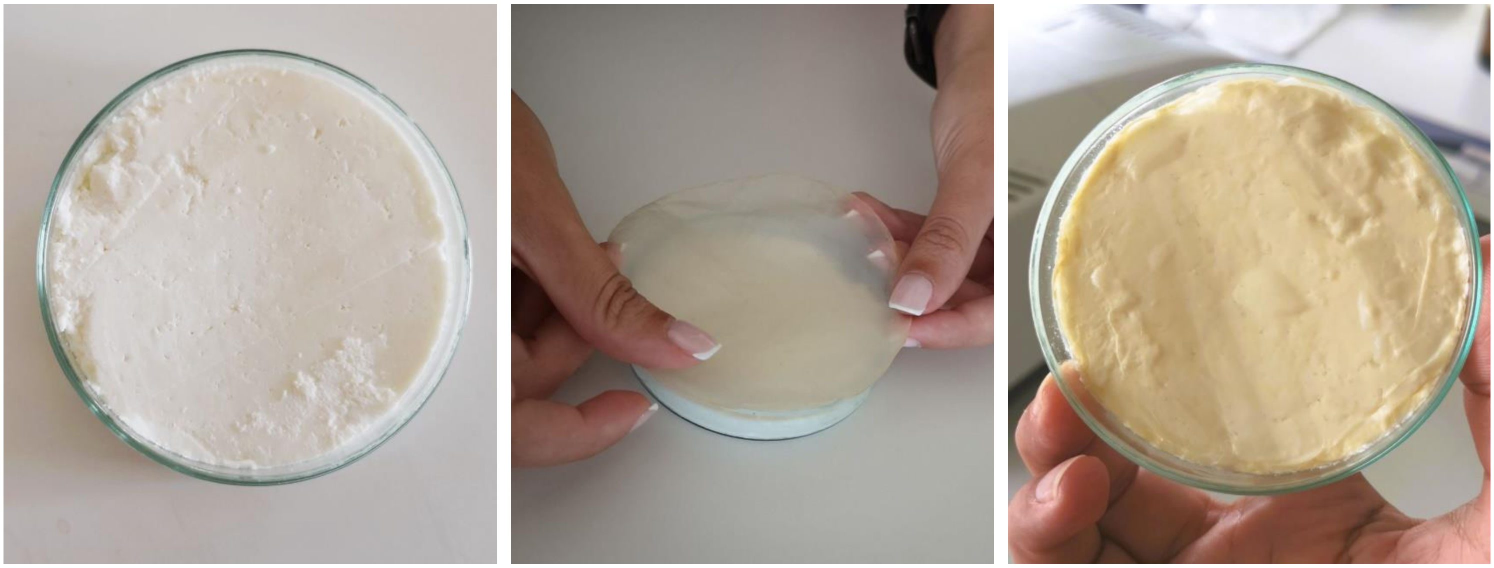

(a) **(b)** **(c)**

Figure S2. (a) White cheese. (b) Cheese surface covering with dried BC film. (c) Cheese covered with dried BC film after 2 months at 4 °C.
